# Polyunsaturated Fatty Acid Composition of Maternal Diet and Erythrocyte Phospholipid Status in Chilean Pregnant Women

**DOI:** 10.3390/nu6114918

**Published:** 2014-11-07

**Authors:** Karla A. Bascuñán, Rodrigo Valenzuela, Rodrigo Chamorro, Alejandra Valencia, Cynthia Barrera, Claudia Puigrredon, Jorge Sandoval, Alfonso Valenzuela

**Affiliations:** 1 Department of Nutrition, Faculty of Medicine, University of Chile, Av. Independencia 1027, Independencia, Santiago 8380453, Chile; E-Mails: rvalenzuelab@med.uchile.cl (R.V.); rodrigochamorro@med.uchile.cl (R.C.); avalencia2@gmail.com (A.V.); cynthia.barrera@gmail.com (C.B.); 2 Obstetrics and Gynecology Department, Clinical Hospital of the University of Chile, Av. Santos Dumont 999, Independencia, Santiago 8380453, Chile; E-Mails: cpuigrredon@hcuch.cl (C.P.); jsandoval@hcuch.cl (J.S.); 3 Lipid Center, Institute of Nutrition and Food Technology (INTA), University of Chile, Av. El Líbano 5524, Macul, Santiago 8380453, Chile; E-Mail: avalenzu@inta.uchile.cl

**Keywords:** pregnancy, dietary intake, *n*-6 and *n*-3 PUFA intake, DHA, fatty acid phospholipids

## Abstract

Chilean diets are characterized by a low supply of *n*-3 polyunsaturated fatty acids (*n*-3 PUFA), which are critical nutrients during pregnancy and lactation, because of their role in brain and visual development. DHA is the most relevant *n*-3 PUFA in this period. We evaluated the dietary *n*-3 PUFA intake and erythrocyte phospholipids *n*-3 PUFA in Chilean pregnant women. Eighty healthy pregnant women (20–36 years old) in the 3rd–6th month of pregnancy were included in the study. Dietary assessment was done applying a food frequency questionnaire, and data were analyzed through the Food Processor SQL^®^ software. Fatty acids of erythrocyte phospholipids were assessed by gas-liquid chromatography. Diet composition was high in saturated fat, low in mono- and PUFA, high in *n*-6 PUFA (linoleic acid) and low in *n*-3 PUFA (alpha-linolenic acid and DHA), with imbalance in the *n*-6/*n*-3 PUFA ratio. Similar results were observed for fatty acids from erythrocyte phospholipids. The sample of Chilean pregnant women showed high consumption of saturated fat and low consumption of *n*-3 PUFA, which is reflected in the low DHA content of erythrocyte phospholipids. Imbalance between *n*-6/*n*-3 PUFA could negatively affect fetal development. New strategies are necessary to improve *n*-3 PUFA intake throughout pregnancy and breast feeding periods. Furthermore, it is necessary to develop dietary interventions to improve the quality of consumed foods with particular emphasis on *n*-3 PUFA.

## 1. Introduction

During the last few decades, considerable scientific interest has been aroused about the beneficial effects of the adequate dietary intake of essential fatty acids. The early life period, *i.e.*, during pregnancy and early postpartum (perinatal period), has been one of the main research foci given that in this period, the composition of dietary fatty acids determines important effects on growth and development [1,2]. *n*-6 and *n*-3 polyunsaturated fatty acids (PUFA) are considered essential dietary nutrients, because mammals do not have the enzymatic capacity to insert a double-bond at the *n*-6 and *n*-3 position of saturated and/or *n*-9 precursors to form linoleic acid (LA, 18:2*n*-6) and alpha-linolenic acid (ALA, 18:3*n*-3), respectively [3,4].

LA and ALA are, in turn, the precursors of a family of fatty acids having a 20- or 22-carbon chain, such as arachidonic acid (AA, 20:4*n*-6) formed from LA and eicosapentaenoic acid (EPA, 20:5*n*-3) and docosahexaenoic acid (DHA, 22:6*n*-3) formed from ALA. AA and DHA are considered the most important metabolic products of LA and ALA, respectively [5,6]. AA and DHA are vital structural components of membrane phospholipids [7]. AA is widely distributed in all cell membrane phospholipids, while DHA is almost exclusively present in a high concentration in membrane phospholipids of cells from the central nervous system (neurons and glial cells) [8]. In humans, the higher rate of AA and DHA accretion occurs during the third trimester of pregnancy and the first two years after delivery [8,9,10]. In this context, both LA and ALA, as precursors of AA and DHA, are essential fatty acids and should be obligatory incorporated throughout the diet. LA is highly available in the Chilean diet, which is characterized as a Western diet. However, ALA is very restrictive, and preformed DHA is only scarcely consumed by our population, because of the low consumption of sea foods, which are the main primary source of this fatty acid [11].

Several studies have shown that pregnancy is a critical period for *n*-3 PUFA intake [12]. Maternal DHA supplementation during pregnancy results in higher scores on visual and neurocognitive tests in children at 12 months of age [13] and improves the early development of visual acuity and other neurodevelopmental indices [14]. Fetal metabolic demand of DHA increases during growth [15] in the last trimester of pregnancy, being the period where DHA accretion to the fetal brain and nervous system reaches its maximum speed [16]. Fetal supply of DHA is provided from maternal circulation at a rate, on average, of 67 mg of *n*-3 PUFA (primarily as DHA) per day [17]. On the other hand, maternal DHA requirements are increased in response to the expansion of red cell mass and placenta and for the accomplishment of the DHA needs of pregnant women [18].

The increased metabolic need of DHA during pregnancy can be compensated for by: (1) dietary intake; (2) increasing the capacity to metabolize ALA to DHA [19]; (3) preferential use of DHA reserves mobilized from adipose tissue [20]; and (4) saving DHA, because of amenorrhea during pregnancy [18,21]. Regarding diet, sources of *n*-3 PUFA are limited, and estimated intakes of EPA and DHA in various populations are below the recommended levels [22]; it is also known that DHA intake of women from industrialized countries is usually low. The average intake of DHA in Western countries is 70 to 200 mg/day [23,24,25,26], but in some cases, the intake is even lower (30–50 mg/day), resulting in women having less than the estimated daily accretion of DHA to the fetus in the third trimester [16]. These observations suggest a potential insufficient dietary intake of DHA for both mother and infant [27]. The assessment of the nutritional status of essential fatty acids during pregnancy is highly relevant, considering its effects on the offspring, infant development and the health of the mother [27].

The objective of this study was to evaluate the dietary supply of *n*-6 and *n*-3 PUFA and the nutritional PUFA status, through erythrocyte membrane phospholipid fatty acid measurement, in a group of healthy pregnant Chilean women. Furthermore, the nutrient intake of women was compared with the specific nutrient recommendations during pregnancy.

## 2. Material and Methods

### 2.1. Subjects

All women recruited to participate in the study were patients of the Obstetrics and Gynecology Department of the Clinical Hospital, University of Chile. Women (*n* = 80), between 20 and 35 years, being in the 3rd–6th month of pregnancy, with a history of previous successful breastfeeding (defined as having a previous child with breastfeeding up to six months) and an absence of any current pathology (hypertension, gestational diabetes, *etc.*) during pregnancy or congenital fetal malformation, were selected. Socioeconomic status was evaluated by the criteria proposed by the European Society for Opinion and Marketing Research (ESOMAR) [28], which includes the assessment of the educational level and current work activity of the individual with the highest income in the household. The study protocol was reviewed and approved by the Institutional Review Board of the Faculty of Medicine, University of Chile (Protocol 073-2011) and by the Ethic Committee of the Clinical Hospital, University of Chile (Protocol 507/11). All information regarding the study was given to each participant who voluntarily agreed to participate and signed the informed consent.

### 2.2. Food Analysis

Dietary evaluation was conducted by trained dietitians of the research group by the application of a food frequency questionnaire. To estimate nutritional composition (energy, macro- and micro-nutrients intake) of the habitual diet (between 3 and 6 months of gestation), mothers were extensively interviewed and asked for all groups of foods consumed during the previous month. In addition to the food frequency questionnaire, dietitians used a photographic “Atlas of Commonly Consumed Foods in Chile” [29], a validated graphic instrument that help to estimate the amount of each food consumed.

Dietary composition data from the food-frequency questionnaire was grouped into 9 food groups (cereals, fruits and vegetables, dairy, meats and eggs, legumes, fish and shellfish, high-lipid foods, oils and fats, sugars and processed foods). Cereals included all cereals and potatoes; fruits and vegetables included all kind of fruits, natural fruits juice and vegetables; dairy products included milk, cheese, fresh cheese and yogurts; meats and eggs included beef, chicken, pork and turkey meat and all their derived products, as well as eggs; fish and shellfish included hake, mackerel, tuna, salmon and shellfish (fresh and frozen); legumes included beans, chickpeas and lentils; high-lipid foods included olives, almonds, peanuts, walnuts, avocado, pistachios and hazelnuts; oils and fat included vegetable oils (mainly sunflower, soybean, canola, grape seed and olive oil) and fats (lard, butter, margarine, mayonnaise and cream); sugars and processed foods included sugar, honey, jam, delicacies, soft drinks, artificial juices, chocolates, cookies and sweet and savory snacks. Fatty acid intake from each food group was calculated as g per 100 g of consumed food, and then the average fatty acid intake was obtained.

Dietary data was analyzed using the software, Food Processor SQL^®^ (ESHA Research, Salem, OR, USA), to calculate energy and nutrient intake. Diet composition was obtained using a database from the USDA National Nutrient Database for Standard Reference, which also contained information from locally-generated nutrient composition data.

### 2.3. Assessment of Nutritional Status

Participants were subjected to a clinical evaluation when incorporated into the study. A physician and a nurse assessed each participant regarding habitual health control under the standard clinical approach for pregnant women. Anthropometric data of weight (kg) and height (m) were assessed to determine body-mass index (BMI, kg/m^2^). BMI was then used to establish maternal nutritional status according to gestational week following the national reference [30]. Energy and nutrient requirements were established according to WHO criteria [31] and recommended dietary intakes according to the American Institute of Medicine, 2001 [32].

### 2.4. Blood Samples

Blood was obtained at the first clinical evaluation at the beginning of the study (between 3 and 6 months of pregnancy) and stored in the presence of butylhydroxytoluene (BHT) as the antioxidant. Erythrocytes were then separated by centrifugation (3000 *g* × for 10 min at 20 °C) and frozen at −80 °C for further analyses.

### 2.5. Fatty Acid Analysis

#### 2.5.1. Lipid Extraction from Erythrocyte Membranes

Quantitative extraction of total lipids from erythrocyte samples was carried out according to Bligh and Dyer [33], in the presence of BHT. Erythrocytes were homogenized in ice-cold chloroform/methanol (2:1 v/v) (containing magnesium chloride 0.5N and 0.01% (w/v) BHT) in an Ultraturrax homogenizer (Janke & Kunkel, Stufen, Germany). Lipids extracted from erythrocyte samples were separated by TLC (aluminum sheets 20 × 20 cm, silica gel 60 F-254, Merck, Santiago, Chile), using the solvent system, hexane/diethyl ether/acetic acid (80:20:1 v/v). After the development of plates and solvent evaporation, lipid spots were visualized by exposing the plates to a Camag UV (250 nm) lamp (Camag, Muttenz, Switzerland)) designed for TLC. The solvent system allows the separation of phospholipids, cholesterol, triacylglycerols and cholesterol esters according to their relative mobility. Spots of individual lipids were scraped from TLC plates and eluted with either diethyl ether or chloroform/methanol (2:1 v/v) [34].

#### 2.5.2. Fatty Acid Methyl Ester (FAME) Synthesis

For fatty acid methyl ester (FAME) formation, phospholipids previously extracted from the silica gel spots with 15 mL of chloroform/methanol/water (10:10:1 v/v) and evaporated under nitrogen stream, were treated with methanolic boron trifluoride (12% methanolic solution) [35] and sodium hydroxide (0.5 N methanolic solution). FAME samples were cooled and extracted with 0.5 mL of hexane.

#### 2.5.3. Gas Chromatography Analysis of FAME

FAME were separated and quantified by gas-liquid chromatography in Hewlett-Packard equipment (model 7890A, CA, USA) using a capillary column (Agilent HP-88, 100 m × 0.250 mm; I.D. 0.25 μm) and a flame ionization detector (FID). The injector temperature was set at 250 °C and the FID temperature at 300 °C. The oven temperature at injection was initially set at 120 °C and was programmed to increase until 220 °C at a rate of 5 °C per min. Hydrogen was utilized as the carrier gas at a flow rate of 35 cm per second in the column, and the inlet split ratio was set at 20:1. The identification and quantification of FAME were achieved by comparing the retention times and the peak area values (%) of the unknown samples to those of a commercial lipid standard (Nu-Chek Prep Inc., Elysian, MN, USA). C23:0 was used as the internal standard (Nu-Chek Prep Inc., Elysian, MN, USA.) using the Hewlett-Packard Chemstation (Palo Alto, CA, USA) data system.

### 2.6. Statistical Analyses

**Figure 1:**
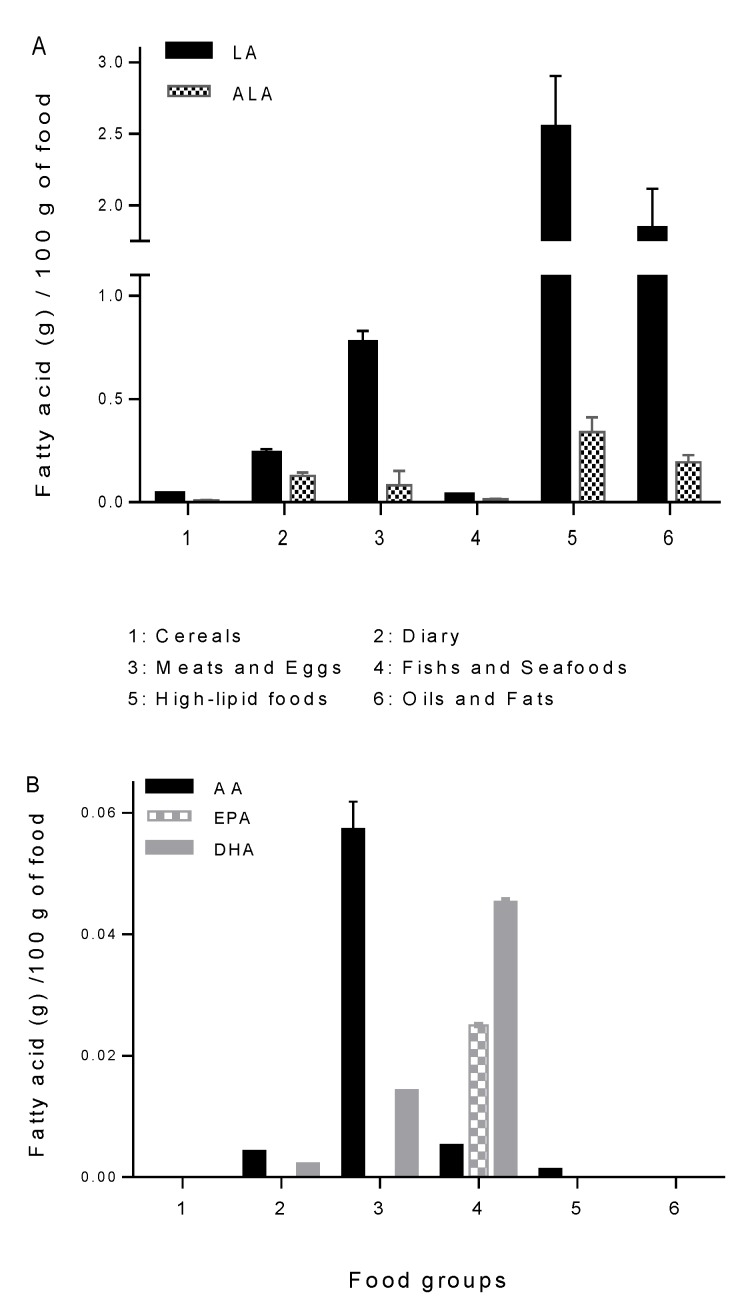
Data are expressed as the mean ± SE and represent the content of each fatty acid (g)/100g of food. Cereals, all cereals and potatoes; dairy products, milk, cheese, fresh cheese and yogurts; meats and eggs, beef, chicken, pork and turkey meat and all their derived products, as well as eggs; fish and shellfish, hake, mackerel, tuna, salmon and shellfish (fresh and frozen); high-lipid foods, olives, almonds, peanuts, walnuts, avocado, pistachios and hazelnuts; oils and fats, vegetable oils (mainly sunflower, soybean, canola, grape seed and olive oil) and fats (lard, butter, margarine, mayonnaise and creams).

Dietary data were checked by contrasting the energy/nutrient intake data composition with dietary questionnaires, identifying potential outliers. In that case, a careful review of each food frequency questionnaire was done. A descriptive analysis was conducted, and the analysis of the variable’s distribution was done using a Shapiro–Wilk test. Results are expressed as the mean ± SD (SE in Figure 1) or the median (interquartile range). To compare dietary nutrient intake with nutrient recommendations, a paired sample *t*-test was used. For all comparisons, statistical significance was set at α level ≤0.05. The statistical software used was SPSS v.15.0 (Chicago, IL, USA) and GraphPad Prism v.5.0 (GraphPad Software, San Diego, CA, USA) for figure processing.

## 3. Results

Table 1 shows the background and anthropometric data of the studied women. The sample was composed of young women (29.3 ± 5.9 years), mainly of medium socioeconomic status (70.9%). The gestational average was 22.6 ± 8.4 weeks. The distribution of nutritional status was 2.6% of women underweight, 51.2% normal weight, 29.5% overweight and 16.7% obese, indicating that over 50% of the sample presented a normal nutritional status.

**Table 1:** Background characteristics.

Variable	(*n* = 80) Mean ± SD
Age (Years)	29.3 ± 5.9
SES	
*High* (%)	13.9
*Medium* (%)	70.9
*Low* (%)	15.2
Preconception Weight (kg)	65.2 ± 10.9
Preconception BMI (kg/m^2^)	25.1 ± 3.6
Weight (kg) ^a^	70.07 ± 10.7
Height (m) ^a^	1.60 ± 0.04
BMI (kg/m^2^)^ a^	26.9 ± 3.4
Gestational Age (Weeks)	22.6 ± 8.4
Nutritional Status	
*Underweight* (%)	2.6
*Normal Weight* (%)	51.2
*Overweight* (%)	29.5
*Obese* (%)	16.7

Data are expressed as the mean ± SD or as a percentage (%); SES, socioeconomic status; BMI, body mass index = kg/m^2^; ^a^ Anthropometric measures were taken at study enrollment.

### 3.1. Dietary Intake

The intake of women was analyzed according to energy intake and macro- and micro-nutrient dietary consumption. Daily nutrient dietary intake is shown in Table 2. Average energy was 2482 ± 670.5 kcal with 15.1%, 52.7% and 32.2% of energy coming from protein, lipids and carbohydrates, respectively. When intake data were compared with nutritional requirements, it was observed that the nutritional adequacy ((intake/requirement) × 100) of energy and macronutrients (carbohydrates, lipids and proteins) was all above the requirements. However, vitamins (folic, choline, thiamine, niacin and E) and minerals (iron, iodine, potassium, magnesium, zinc and copper) were under the recommended level. The intake of vitamins A, riboflavin, vitamins C and vitamins B12 and minerals (calcium, phosphorus and sodium) were above the recommended level (Table 2).

Daily fat and relevant *n*-6 and *n*-3 fatty acid intake are shown in Table 3. Total fat intake was over the recommendations. When saturated and unsaturated fat intakes were compared, it was observed that saturated fat was above the recommendations and total unsaturated fat (mono- and polyunsaturated) was under the recommendations. It is remarkable that the insufficient intake of ALA, EPA and DHA generated a high imbalance of the *n*-6/*n*-3 ratio (Table 3).

Fatty acid intake according to dietary food group is shown in Table 4. Fatty acid intake was analyzed and separated into nine dietary groups. Regarding total fat intake, higher fat consumption was obtained from oils and fats, followed by meats and eggs, cereals, high-lipid foods and dairy. Saturated fatty acid (SAFA) consumption was supplied by dairy, followed by meats and eggs and oils and fats. Monounsaturated fatty acids (MUFA) came from high-lipid foods, followed by oils and fats, meats and eggs, dairy and cereals. Regarding total PUFA intake, the higher supply came from oils and fats, followed by high-lipid foods, cereals, meats and eggs and dairy. The contribution of the different food groups to the dietary intake of *n*-6 and *n*-3 PUFA is shown in Figure 1A (LA and ALA) and Figure 1B (AA, EPA and DHA). It can be observed that the supply of LA was from high-lipid foods, followed by oils and fats and meats. ALA was notably less consumed than LA (Figure 1A). AA was provided mainly by meats, and EPA and DHA came almost exclusively from fish.

**Table 2:** Daily energy and nutrient dietary intake.

Energy/Nutrients	Intake	Requirement/RDA ^a^	Adequacy (%) ^b^	*p*-Value
Energy (kcal)	2482.0 ± 670.5	1835.8 ± 79.3	135	0.01
Protein (g)	94.0 ± 29.2	59.4 ± 3.4	158	0.001
Carbohydrate (g)	326.9 ± 106.4	238.3 ± 53.1	137	0.001
Fat (g)	88.8 ± 31.9	61.2 ± 2.6	145	0.001
Fiber (g)	30.5 ± 12.1	28	108	0.07
Cholesterol (mg) ^c^	266.5 ± 104.4	-	-	-
Trans Fatty Acid (g) ^c^	1.3 ± 1.5	-	-	-
Iron (mg)	14.4 ± 6.1	27	53	0.01
Folic Acid (μg) ^d^	408.7 ± 261.9	600	68	0.01
Choline (mg)	217.2 ± 98.5	450	48	0.01
Iodine (μg) ^e^	74.8 ± 67.2	220	33	0.01
Calcium (mg)	1111.9 ± 511.3	1000	119	0.05
Vitamin A (RAE) ^f^	905 ± 513.5	770	117	0.02
Thiamin (mg)	1.2 ± 0.7	1.4	85	0.01
Riboflavin (mg)	1.5 ± 0.7	1.4	107	0.19
Niacin (mg)	11.6 ± 6.0	18	64	0.01
Vitamin C (mg)	196.0 ± 106.7	85	230	0.01
Vitamin E (α-tocopherol, mg)	5.8 ± 3.5	15	38	0.01
Vitamin B12 (μg)	4.3 ± 2.6	2.6	165	0.01
Phosphorus (mg)	985.1 ± 421.8	700	140	0.01
Sodium (g)	2.76 ± 1.9	1.5	164	0.01
Potassium (g)	2.9 ± 1.1	4.7	61	0.01
Magnesium (mg)	214.8 ± 101.8	350	61	0.01
Zinc (mg)	8.4 ± 4.63	11	76	0.01
Copper (μg)	914.4 ± 462.8	1000	91	0.10

Data are expressed as the mean ± SD; ^a^ RDA: Recommended Dietary Allowance, according to the Institute of Medicine, National Academies, USA [32]; ^b^ adequacy: (nutrient intake/nutrient daily recommendation) × 100; ^c^ for this nutrient, the proposed recommendation is “as low as possible while consuming a nutritionally adequate diet”; ^d^ folic acid intake does not include intake from fortified products (wheat flour); ^e^ iodine intake does not include intake from fortified products (salt); ^f^ RAE, retinol activity equivalent; 1 RAE = 1 mg retinol.

**Table 3:** Daily fat and relevant *n*-6 and *n*-3 fatty acid dietary intake.

Nutrients	Intake	RDA ^a^	Adequacy (%) ^b^	*p*-Value ^c^
Total Fat (g)	88.8 ± 31.9	61.2 ± 2.6	145	0.001
Saturated Fat (g)	26.7 ± 11.7	15.6 ± 1.6	171	0.001
Monounsaturated Fat (g)	23.3 ± 10.5	29.0 ± 1.1	80	0.001
Polyunsaturated Fat (g)	16.3 ± 9.1	22.3 ± 0.8	73	0.001
LA (g) *	4.4 (2.8–6.7) ^c^	13.0	45	0.001
ALA (g) *	0.6 (0.4–1.0) ^c^	1.4	67	0.001
ARA (mg) *	60.0 (40–90) ^c^	800	7.5	0.001
EPA (mg) *	10.0 (0–50) ^c^	100	31	0.001
DHA (mg) *	40.0 (10–100) ^c^	200	33	0.001
*n*-6/*n*-3 Ratio **	8.1 ± 6.1	-	-	-

Data are expressed as the mean ± SD, unless otherwise specified; ^a^ RDA: Recommended Dietary Allowance (RDA) according to the Institute of Medicine, National Academies, USA [32]; ^b^ adequacy: ((nutrient intake/nutrient daily recommendation) × 100); ^c^ for a comparison between mean intake and RDA proposed values; * Data expressed as the median (interquartile range); ** There is no RDA for this ratio; - no adequacy is shown; LA, linoleic acid; ALA, alpha-linolenic acid; ARA, arachidonic acid; EPA, eicosapentaenoic acid; DHA, docosahexaenoic acid.

**Table 4:** Fatty acid intake according to food groups.

Food Groups	Total Fat (g)	Total SAFA (g)	Total MUFA (g)	Total PUFA (g)
Cereals	9.7 (7.0–12.7)	1.7 (1.3–2.4)	1.0 (1.5–2.2)	1.2 (0.7–1.8)
Fruits and Vegetables	1.2 (0.7–1.6)	0.1 (0.07–0.17)	0.05 (0.02–0.07)	0.2 (0.1–0.3)
Dairy	8.4 (4.4–18.0)	4.9 (2.8–10.8)	1.9 (0.9–4.3)	0.2 (0.1–0.6)
Meats and Eggs	12.5 (8.3–20.4)	4.4 (2.8–6.7)	2.8 (1.8–5.7)	0.9 (0.5–1.2)
Fish and Seafood	0.7 (0.2–1.6)	0.17 (0.008–0.064)	0.2 (0.01–0.4)	0.1 (0.01–0.2)
Legumes	0.18 (0.4–0.39)	0.01 (0.006–0.04)	0.02 (0.007–0.06)	0.1 (0.02–0.2)
High-Lipid Foods	9.9 (3.7–14.4)	1.2 (0.5–1.8)	5.0 (2.5–7.6)	1.6 (0.6–3.7)
Oils and Fats	25 (4.9–36.2)	4.2 (2.2–6.4)	4.7 (3.2–9.1)	9.6 (3.9–15.0)
Sugar, Alcohol and Processed Foods	3.4 (0.84–8.1)	0.9 (0.2–3.0)	0.01 (0–0.02)	0.004 (0–0.09)

Data are expressed as the median (interquartile range); SAFA, saturated fatty acids; MUFA, monounsaturated fatty acids; PUFA, polyunsaturated fatty acids; Cereals, all cereals and potatoes; fruits and vegetables, fruits, natural fruits juice and vegetables; dairy products, milk, cheese, fresh cheese and yogurts; meats and eggs, beef, chicken, pork and turkey meat and all of their derived products, as well as eggs; fish and shellfish, hake, mackerel, tuna, salmon and shellfish (fresh and frozen);legumes, beans, chickpeas and lentils; high-lipid foods, olives, almonds, peanuts, walnuts, avocado, pistachios and hazelnuts; oils and fats, vegetable oils (mainly sunflower, soybean, canola, grape seed and olive oil) and fats (lard, butter, margarine, mayonnaise and creams); sugars and processed foods, sugar, honey, jam, delicacies, soft drinks, artificial juices, chocolates, cookies and sweet and savory snacks.

### 3.2. Fatty Acid Composition of Erythrocyte Phospholipids

The fatty acid composition of phospholipids extracted from erythrocyte membranes is shown in Table 5. Total SAFA was the predominant fatty acid, followed by total PUFA and total MUFA. *n*-6 PUFA was almost four times the *n*-3 PUFA, LA and AA being the predominant fatty acids. DHA is the predominant *n*-3 PUFA, the values for ALA and EPA being closely similar. Table 5 also includes the results from the literature obtained for pregnant women of three different countries and continents (China, Belgium and the United States of America (USA)). The analysis of this information compared with our results is found in the Discussion section.

**Table 5:** Fatty acid composition of maternal erythrocyte membrane phospholipids

Fatty Acids ^a^	Chilean Women ^b^	Chinese Women ^c^	Belgium Women ^d^	USA Women ^e^
Total SAFA	52.2 ± 2.8	46.4 (44.7–47.2)	46.0 ± 3.3	*
Total MUFA	13.3 ± 1.5	14.5 ± 3.5	12.7 ± 1.3	*
Total PUFA	35.4 ± 3.3	36.6 (34.1–38.7)	38.2 ± 3.5	*
Total *n*-6 PUFA	28.6 ± 3.6	26.5 (24.6–28.3)	*	27.91 ± 5.39
Total *n*-3 PUFA	6.8 ± 1.0	9.8 (8.6–11.8)	*	6.96 ± 2.27
18:2, *n*-6 (LA)	14.6 ± 3.4	15.0 ± 4.6	19.1 ± 3.2	9.0 ± 1.49
18:3, *n*-3 (ALA)	1.2 ± 0.4	*	0.22 ± 0.14	0.13 ± 0.06
20:4, *n*-6 (AA)	13.2 ± 1.8	7.3 (5.7–8.5)	8.4 ± 1.8	13.09 ± 3.3
20:5, *n*-3 (EPA)	1.6 ± 0.5	1.9 (1.7–2.2)	0.50 ± 0.31	0.30 ± 0.17
22:6, *n*-3 (DHA)	3.6 ± 0.6	5.6 (4.1–8.1)	4.8 ± 1.3	4.74 ± 1.68
*n*-6/*n*-3 PUFA Ratio	4.3 ± 1.0	2.6 (2.1–3.2)	*	4.71 ± 2.8

^a^ Fatty acids are expressed as g per 100 g fatty acid methyl ester (FAME); ^b^ Data are expressed as the mean ± SD; SAFA, saturated fatty acids; MUFA, monounsaturated fatty acids; PUFA, polyunsaturated fatty acids; LA, linolenic acid; ALA, alpha-linolenic acid; AA, arachidonic acid; EPA, eicosapentaenoic acid; DHA, docosahexaenoic acid; SAFA includes: 6:0, 8:0, 10:0, 12:0, 14:0, 16:0, 18:0, 20:0, 22:0 and 24:0; MUFA includes 14:1 *n*-5, 16:1 *n*-7 and 18:1 *n*-9; PUFA includes 18:2 *n*-6, 18:3 *n*-3, 20:4 *n*-6, 20:5 *n*-3, 22:5 *n*-3 and 22:6 *n*-3; *n*-6/*n*-3 PUFA ratio: 20:4 *n*-6/(20:5 *n*-3 + 22:5 *n*-3 + 22:6 *n*-3); ^c^ Data from [22]; ^d^ Data from [36]; ^e^ Data from [37]; *: no data was available in this study.

## 4. Discussion

This study evaluated the dietary composition consumed by a sample of Chilean pregnant women with emphasis on their fatty acid intake and its association with the fatty acid profile of erythrocyte membrane phospholipids. Energy and macronutrient (carbohydrate, protein and fat) intake exceeded daily recommendations, but several vitamins and minerals were considerably under the recommended daily intake. The overconsumption of total fat and, especially, saturated fat in conjunction with an excessive intake of total *n*-6 PUFA (mainly LA) *versus* a lower intake of *n*-3 PUFA, particularly ALA, EPA and DHA, was observed. Considering these results, it is interesting to observe that LA (precursor of AA) intake is under the recommendation; however, this is not reflected in the AA content of erythrocyte membrane phospholipids (Table 5). ALA is also under the recommendation, and as a precursor of EPA and DHA, these *n*-3 PUFA show values far lower than the minimal recommendation. This suggest that the nutritional imbalance observed in the sample is due to either an efficient conversion of LA to AA (in spite of the low LA consumption), a very low conversion of ALA to DHA or to both effects [38]. This dietary imbalance is also observed in the fatty acid composition of erythrocyte phospholipids, which is considered the better marker for the nutritional fatty acid status [39].

The imbalance found between *n*-6 and *n*-3 PUFA (*n*-6/*n*-3 ratio) in the diet and in membrane phospholipids in pregnant women prompts a warning considering the effects of this imbalance in the human gestational period. During pregnancy, the rate of growth and development of the central nervous system is higher in the final stage (third trimester) and in the early postnatal period [17]. There is evidence demonstrating that the accumulation of DHA in the membrane of neuronal cells during pregnancy and early infancy reaches 50 mg/kg/month, whereas AA accumulates at a rate of 400 mg/kg/month, approximately [40,41,42]. Dietary intake of *n*-3 PUFA is associated with the enrichment of *n*-3 PUFA into the nervous system in pregnancy and the first month of extra-uterine life, strongly impacting infant neurodevelopment [43,44,45,46]. The low intake of DHA and the diminished status of this fatty acid in membrane phospholipids of maternal erythrocytes could determine a lower supply of the fatty acid to fetal tissues and, in turn, the depletion of maternal stores, as has been suggested [47]. Furthermore, the low intake of *n*-3 PUFA and the poor *n*-3 PUFA maternal status, particularly of DHA, could eventually determine lower neurocognitive development in infants [48].

The characterization of dietary fat intake of pregnant women based on the classification according to food groups (Table 2 and Table 4) allowed us to determine the food source of total fat and the principal fatty acids in the diet. In relation to PUFA, the major food groups contributing to LA intake were the high-lipid foods, oils and fats and meats and eggs, whereas for ALA, these were high-lipids foods, oils and fats and dairy products. As expected, the highest proportion of EPA and DHA came from the group of fish and seafood, followed by the minimal contribution of meats and dairy products, only in the case of DHA. Regarding AA, the largest proportion came from meats, eggs and their derivatives, with a low contribution of other groups, such as dairy and seafood (Figure 1).

The high proportion of LA in the diet may be related to the high intake of vegetable oils, such as sunflower and soybean oils, and of fat from butter, margarine, meats and its derived products, all foods that are preferably consumed in the Chilean and occidental diet [49]. The low ALA intake came primarily from some oily seeds (walnuts, almonds) and vegetable oils (soybean and canola), with a lower proportion from dairy products. The low EPA and DHA intake observed in these pregnant women is a consequence of the low consumption of fish and shellfish, because this food group is the main and largest contributor to both fatty acids. The average daily intake of DHA was far below the recommended level, approximately allowing only 30% of the recommendation [50,51]. Results from other countries, included in Table 5, are very interesting. Regarding the comparison of fatty acid profile in membrane phospholipids with the other populations, the characteristics of our sample are similar to those reported in Belgium and USA pregnant women [37], pointing to a low *n*-3 PUFA and high *n*-6 PUFA content and, consequently, a higher *n*-6/*n*-3 ratio (compared only to USA women), contrary to what was reported in Chinese pregnant women [22]. Although these results are not strictly comparable, it is possible to establish that pregnant Chilean women have the lowest DHA concentration in the phospholipids of erythrocyte membranes.

The low intake of *n*-3 PUFA and the excessive intake of LA indicate that the average diet of Chilean pregnant woman has an imbalance towards the family of *n*-6 PUFA. Because of this reason, a complementary feeding program was available from 2009 in the country, consisting of a dry powdered milk product, which provides 90 mg of DHA per 200 mL [52], which is cost-free supplied to pregnant and lactating woman through the Chilean Primary Care Service system. The acceptability of the product was evaluated in pregnant women establishing that some modification of the milk product must be introduced [53]. It is also necessary to rethink the nutritional strategies to reduce saturated fat consumption, aiming to prevent chronically non-transmissible diseases [54] and to increase the *n*-3 PUFA consumption in this group and in the general population. The dietary imbalance observed in the intake of other important micronutrients during pregnancy, such as choline, iron, zinc and others vitamins or minerals, could negatively affect optimal infant development and maternal nutritional status [55,56], indicating that urgent dietary strategies in this group are needed.

Regarding fatty acids, although obviously the recommendation is to increase the consumption of seafood, the availability of these products is low, of high cost and concerns have arisen regarding contaminants [57]. The development of new food alternatives is essential to meet the daily recommendation of *n*-3 PUFA, specifically DHA, particularly in pregnant and lactating women. The traditional strategy to supplement DHA, such as fish oil capsules or emulsions, microalgae oil, *etc.*, has proven to be beneficial, particularly in improving neurological development [39]. The promotion of the consumption of vegetable oils with a high content of ALA (such as canola, chia, *Camelina*, flaxseed oil) [58,59] could be a new and interesting strategy to provide *n*-3 PUFA during this important physiological period [60]. It was demonstrated that consumption of canola oil (10% ALA) by Chilean women during pregnancy reduced the risk of premature delivery and improved the birth weight of infants [61].

## 5. Conclusions

The sample of Chilean pregnant women showed a high consumption of saturated fat and low consumption of *n*-3 PUFA (ALA, EPA and DHA), which is reflected in the low DHA content of phospholipids from erythrocyte membranes, which are considered a good marker of the nutritional status of this fatty acid. The imbalance of *n*-6/*n*-3 PUFA and the low content of erythrocyte DHA, which results in a restrictive fetal supply of the fatty acid, could negatively affect fetal development with future possible effects on cognitive performance, such as the learning and memory skills of children. It is critical to evaluate new strategies aiming to improve the intake of *n*-3 PUFA throughout pregnancy and the breast feeding periods. It is necessary to develop dietary interventions aimed at improving the quality of consumed foods, with particular emphasis on specific fatty acids, such as *n*-3 PUFA.
